# Monitoring longitudinal disease progression in a novel murine *Kit* tumor model using high-field MRI

**DOI:** 10.1038/s41598-022-17880-y

**Published:** 2022-08-26

**Authors:** Markus Kraiger, Tanja Klein-Rodewald, Birgit Rathkolb, Julia Calzada-Wack, Adrián Sanz-Moreno, Helmut Fuchs, Eckhard Wolf, Valérie Gailus-Durner, Martin Hrabě de Angelis

**Affiliations:** 1 Institute of Experimental Genetics, German Mouse Clinic, Helmholtz Zentrum München, German Research Center for Environmental Health, Neuherberg, Germany; 2 German Center for Diabetes Research, Helmholtz Zentrum München, German Research Center for Environmental Health, Neuherberg, Germany; 3 Institute of Molecular Animal Breeding and Biotechnology, Gene Center, Ludwig-Maximilians-Universität München, Munich, Germany; 4 Chair of Experimental Genetics, TUM School of Life Sciences, Technische Universität München, Freising, Germany

**Keywords:** Breast cancer, Cancer imaging, Cancer models, Gastrointestinal cancer

## Abstract

Animal models are an indispensable platform used in various research disciplines, enabling, for example, studies of basic biological mechanisms, pathological processes and new therapeutic interventions. In this study, we applied magnetic resonance imaging (MRI) to characterize the clinical picture of a novel N-ethyl-N-nitrosourea-induced *Kit*-mutant mouse in vivo. Seven C3H *Kit*^N824K/WT^ mutant animals each of both sexes and their littermates were monitored every other month for a period of twelve months. MRI relaxometry data of hematopoietic bone marrow and splenic tissue as well as high-resolution images of the gastrointestinal organs were acquired. Compared with controls, the mutants showed a dynamic change in the shape and volume of the cecum and enlarged Peyer´s patches were identified throughout the entire study. Mammary tumors were observed in the majority of mutant females and were first detected at eight months of age. Using relaxation measurements, a substantial decrease in longitudinal relaxation times in hematopoietic tissue was detected in mutants at one year of age. In contrast, transverse relaxation time of splenic tissue showed no differences between genotypes, except in two mutant mice, one of which had leukemia and the other hemangioma. In this study, in vivo MRI was used for the first time to thoroughly characterize the evolution of systemic manifestations of a novel *Kit*-induced tumor model and to document the observable organ-specific disease cascade.

## Introduction

A broad variety of neoplasms, such as gastrointestinal stromal tumors (GIST), acute myeloid leukemia (AML), mastocytosis, germinomas and melanomas have been shown to be induced by mutations in the *KIT* gene^1,2^. Mouse models offer an essential platform for elucidating molecular processes and for identifying genetic elements involved in susceptibility and disease progression of cancer. Mouse lines carrying different activating mutations in the *Kit* gene are available^3–5^. Models with constitutive KIT activity, microcytic polycythemia, abnormal cecum morphology, development of GIST, melanoma or mastocytosis have been described^6–10^. Phenotypes observed in different models are similar but apparently differ concerning severity depending on the causative mutation and the genotype. In vivo imaging techniques that can distinguish between different courses are highly desirable because they enable the study of different risk factors even in small numbers of experimental animals.

In the past our laboratory has established an N-ethyl-N-nitrosourea-induced (ENU) novel mouse model (tentative laboratory name MVD013) carrying the N824K mutation in the *Kit* gene. Mutant mice showed hematological phenotypes, morphological alterations of gastrointestinal organs, hyperplasia of interstitial cells of Cajal (ICC) as well as development of GIST and mammary carcinoma. Furthermore, hints towards significant effects of the genetic background on disease progression were observed by analyzing mutants generated on a hybrid background. The detailed phenotypic characterization is reported in the accompanying paper^11^. In-vivo experiments are expected to facilitate studies concerning the impact of potential risk factors such as environmental conditions, diet and genetic background. In particular, of great importance are effects on observable phenotypes and malignancy^12,13^. In-vivo imaging and monitoring techniques are appropriately suited to characterize such interactions on phenotypes and on the malignancy simultaneously. Further, the resulting temporal characterization of disease progression is complementing terminal histopathological findings derived at distinct time points.

In clinical routine the standard method for assessing GIST in-vivo is computed tomography (CT). Alternatively, magnetic resonance imaging (MRI) as a non-invasive and non-ionizing imaging method has opened up a wide spectrum of diagnostic imaging applications. In the field of oncology MRI has been recognized as a valuable modality for visualizing tumor morphology and quantifying growth rates^13–16^. Further, MR relaxometry approaches enable the in-vivo estimation of iron load, and are thus capable of evaluating alterations of hematopoietic tissues related to the phenotype of microcytic polycythemia^17,18^. In the situation of hematologic malignancies such as leukemia, MR relaxation measurement and MR spectroscopy (MRS) studies have proven to be a sensitive method assessing bone marrow alterations^19–21^. Hence, MRI based monitoring enables the acquisition of morphological as well as relaxation data, and as such it is regarded the method of choice for the actual animal model.

In the current work, longitudinal MRI was applied for the characterization of temporal patterns of distinct phenotypes induced in a novel murine *Kit* mutant cancer model. MR relaxometry of hematopoietic tissue in vertebral bone marrow and spleen, and morphological analysis of gastrointestinal (GI) organs were performed. Subsequently, selected imaging findings were correlated with macroscopic and histopathological analysis.

## Results

### Body weight

**Figure 1:**
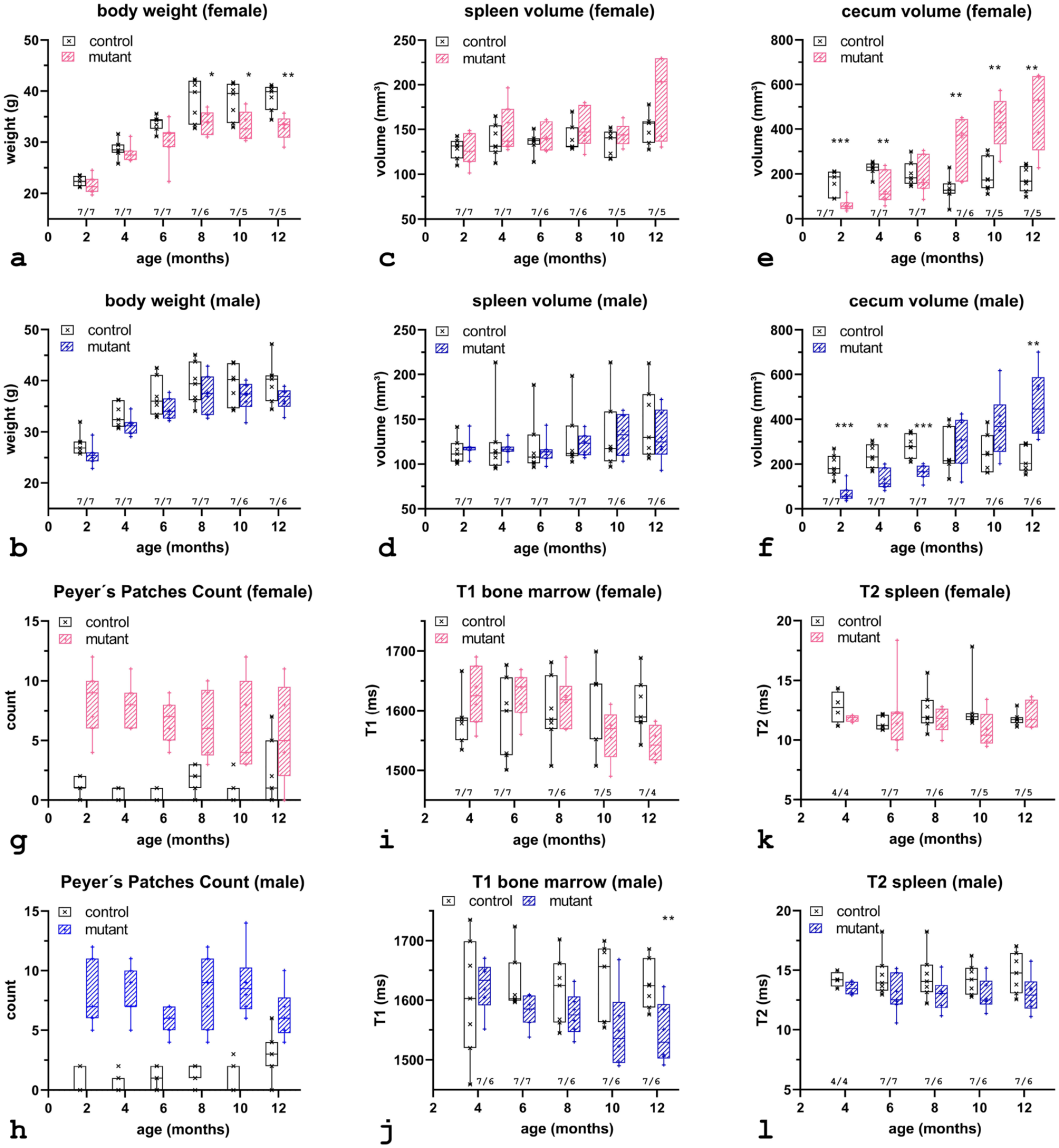
*Body weight* (**a**, **b**) significantly reduced weight was observed for the mutant females (**a**) initially detectable at the age of 8 months. *Splenic volume* (**c**, **d**) no genotype dependent difference was noticed; a sex-specific difference was detected at four and eight months. At the third measurement point data of one female bearing splenic cancer was excluded. *Cecal volume* (**e**, **f**) a temporal varying phenotype was observed for the cecal volume in mutants. The progression correlated with the form of the cecum, the initial decreased volume turned into a significant increase at later age. *Peyer’s Patches* (**g**, **h**) analysis of the MR images revealed prominent Peyer’s patches located inside the cecum. Manual enumeration revealed that, on average, up to five times more nodules were present in the mutants. *T1 relaxometry* (**i**, **j**) revealed possible changes in the cell composition of the hematopoietic tissue of the mutants with increasing age. Compared with controls, a trend toward T1 shortening was observed. *T2 relaxometry* (**k, l**) no genotype dependent difference was observed. However, T2 times were significantly longer in males, indicating a higher iron load in females. Markers representing individual data samples, bar indication median value; animal numbers are given below the box plots; *, *p* ≤ 0.05; **, *p* ≤ 0.01.

A comparison between the genotypes revealed that starting from eight months till the end of the study mutants were lighter than controls, reaching statistical significance only in females after eight months (8 months: *p* < 0.05; 10 months: *p* < 0.05; 12 months: *p* < 0.01). Mutant males were significantly heavier than females, reaching statistical significance at the first three time points (2 months: *p* < 0.01; 4 months: *p* < 0.01; 6 months: *p* < 0.05) and at one year of age (*p* < 0.05). In the control group, males were significantly heavier at the first two time points (2 months: *p* < 0.001; 4 months: *p* < 0.01). The weight curves over the period of investigation are given in Fig. 1a,b, the data is presented in Supplementary Table 1 online. In each group, mean body weight increased steadily during the first four measurement time points, while it remained almost constant at ten and twelve months of age. One female mutant experienced weight loss between the measurement time points at four and six months of age. Based on MRI data, this female was found to have a splenic tumor, thus reaching humane endpoint.

### Spleen volume and morphology

Similar splenic volumes were found in mutant and control mice. It should be noted that the volumes of females were larger than those of males from four months of age. In the mutants, the differences were substantial at the age of four, six, eight and twelve months. However, no significant difference between the two genotypes was detected. The splenic volumes for each time point are given in Fig. 1c,d, the corresponding data can be found as Supplementary Table 1 online. No morphological differences between mutant and control mice were observed, except splenic enlargement due to tumor progression in one female and one male mutant. The wider range in the male control group reflects the impact of a single animal which exhibited almost doubled splenic volume. In the mutant female group an increase of the mean and the standard deviation can be seen at the last time point. This reflects the circumstance that in three mutants the volume at the age from ten to twelve months increased by nearly forty-five percent.

### Cecum volume and morphology

**Figure 2:**
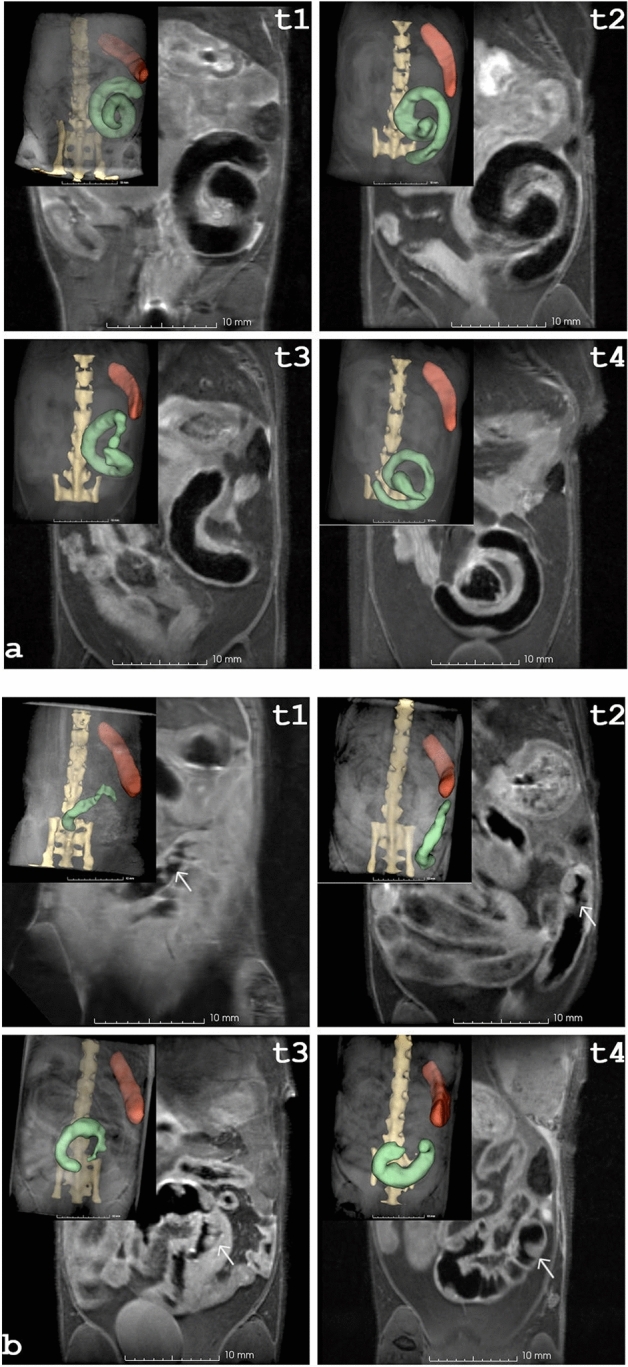
Comparison of cecal development in a female control mouse (**a**) and a female mutant mouse (**b**) along the first four time points (t1–t4). The spine is colored yellow, the spleen is shown in red and the cecum is colored green. At two (t1) and four (t2) months of age, the cecum of the mutant had an elongated shape, whereas the cecum in the control group was arranged in a coiled shape. At later time points, the shape of the mutant cecum resembled the anatomy of the control. Peyer´s patches were clearly visible in the inner wall of the mutant cecum (marked by white arrow in **b**).

**Figure 3:**
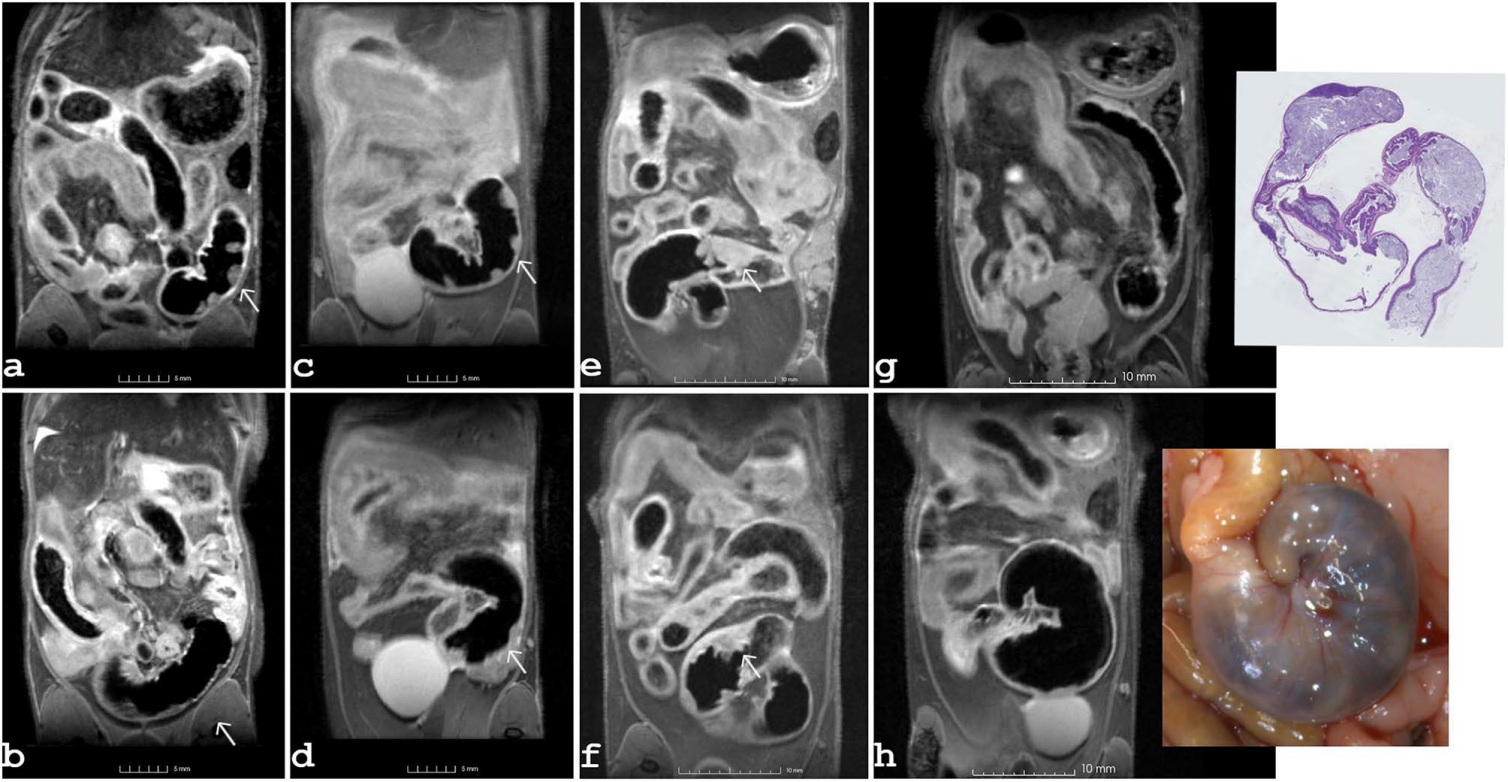
Examples of the development of the Peyer´s patches are given for a mutant female (**a**, **b**) and a male mutant (**c**, **d**) at an age of six and eight months. A comparison between the first and the second row illustrates the decreasing visibility of the patches at later time points. A striking thickening of the cecal wall was noted with increasing age, as observed, for example, in two female mutants at 12 months of age (**e**, **f**) (thickening is indicated by an arrow). (**g**) an example of the observed Peyer’s patches detected in a control along with associated histology. (**h**) coronal image showing the enlarged cecum of a mutant, and the corresponding macro image.

Three major morphological findings were identified distinguishing mutant from control cecum. The mutant animals exhibited an atypically shaped cecum compared with the normal morphology of control mice. Figure 2 shows an example of the cecal development of a female control (Fig. 2a) and a female mutant (Fig. 2b) during the first eight months (t1–t4). Mutant mice exhibited a small cecum at two months of age. With time the shape evolved similar to the form of the controls, but with increased cecal volume due to an increased diameter. In control animals the cecal volume increased slightly between the first two time points and then remained nearly stable, while a continuous increase was observed for mutants. Comparison of averaged volumes shows that in the first six months, controls, with the exception of six-month-old females, had significantly larger volumes (f/m 2 months : *p* < 0.001; f/m 4 months: *p* < 0.01; m 6 months: *p* < 0.001) than mutants (Fig. 1e,f). From eight months onwards this situation changed leading to larger volumes in mutants than in controls, data summarized in Supplementary Table 1. Second, prominent Peyer´s patches were detected that extended into the lumen of the cecum. Based on the MRI data, the detectable patches in the control groups varied from none to three, about five times more nodules were identified in the mutant groups (Fig. 1g,h, Supplementary Table 2). The prominent Peyer’s patches, the “immune sensors” of the intestine^22^, exhibited enlarged dimensions in the mutants (Fig. 2b). With increasing cecal mass these enlarged lymphoid nodules were less visible at later time points. This decline in visibility is exemplary shown for a female (Fig. 3a,b) and for a male (Fig. 3c,d) at the age of six versus eight months. The increased volume of the cecum and loss of visibility of Peyer's patches were due to congestion of the cecum, as shown by comparison with the pathohistologic findings (Supplementary Fig. 1). A third finding observed with increasing age was a conspicuous thickening of the cecal wall. This correlates with the hyperplasia of ICC detected in the histopathology. An example is given in Fig. 3e,f, which shows two female mutants at the age of twelve months that exhibited marked thickening of the wall near the ileocecal junction. Figure 3g shows exemplary MRI data together with the corresponding histology of the cecum of a male control animal at the age of one year, while Fig. 3h depicts the situation of an enlarged congested cecum in a one-year-old mutant male along with the macroscopic pathology photo. An exemplary comparison with the histopathologic finding is shown in Supplementary Fig. 2.

### Tumors

**Figure 4:**
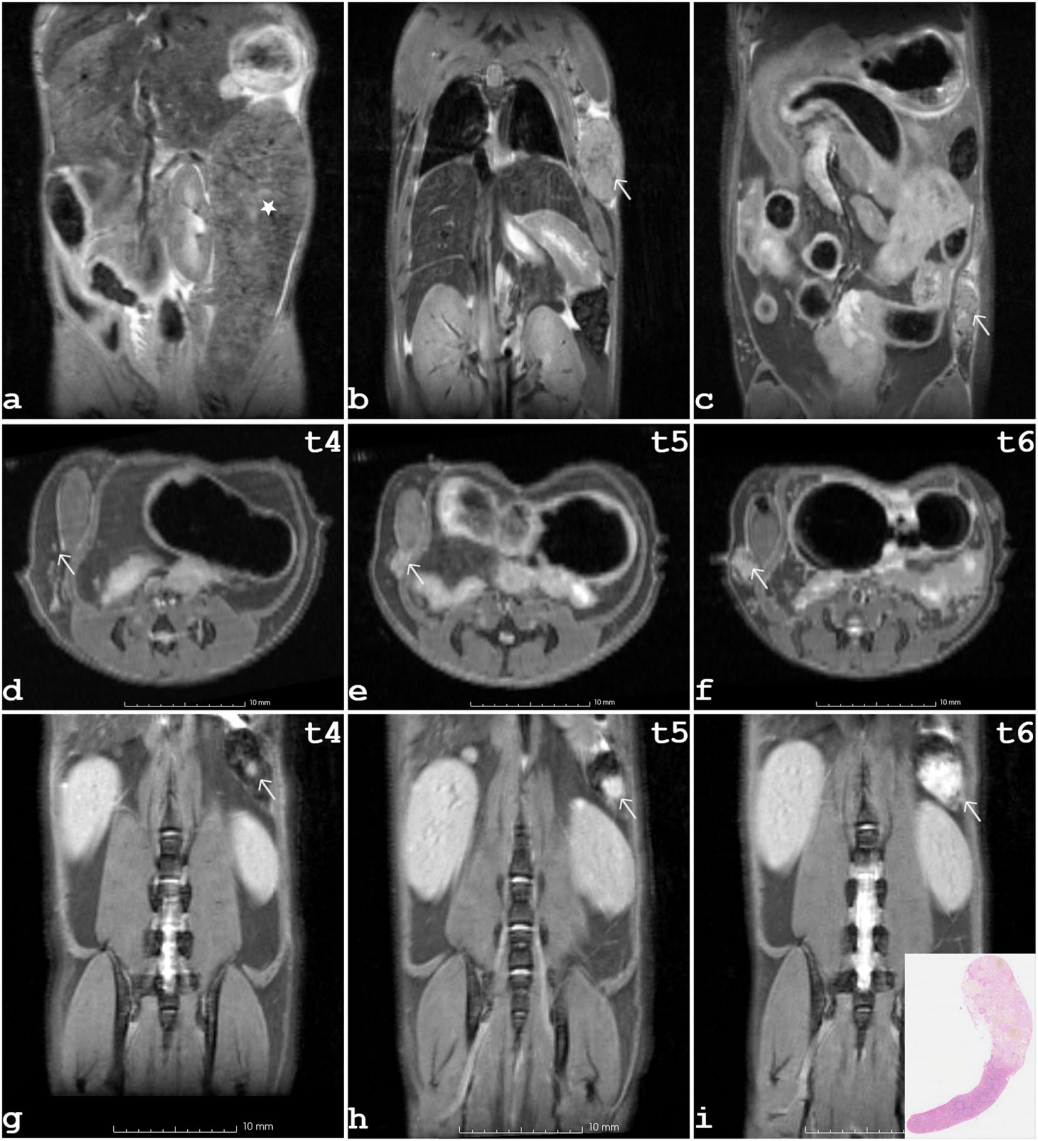
Representative tumor findings in mutant mice. At the third observation time point, a female mutant was found to have a large spleen (**a**). In (**b**) tumorous tissue was observed in the axillary region of an 8-month-old female. In (**c**) cancerous tissue is located in the pelvic region of a one-year-old female. (**d**–**i**) panel showing 2D fs-RARE data, starting at eight months of age, tracking cancer development in two mutant individuals over a six-month period. A mammary tumor is shown in (**d–f**), and a splenic hemangioma (tumor indicated by white arrow) is shown in (**g–i**) with the corresponding histologic finding as an insert in (**i**).

An overview of the monitored tumors, their anatomical location and the estimated volumes is given in Table 1. The earliest MRI observation of a tumor was made at an age of six months. A female mutant mouse showed dramatically increased splenic volume, the spleen covering almost one third of the abdominal cavity. The extent is visible on a representative slice of the 2D fs-RARE data given in Fig. 4a. A splenic volume of 1547 mm^3^ was estimated, although the dimension of the spleen exceeded the field of view (FOV). On histological analysis, this animal was diagnosed with leukemia. At the age of eight months mammary cancer was detected in another two female mutants. One female bearing tumorous tissue in the axillary region, as shown in Fig. 4b. For the second female mutant a hyper-intense spot on the T2-weighted fs-RARE data indicated onset of tumorous tissue in the pelvic region. Further, a hyper-intense spot within the spleen was noticeable on the fs-RARE images of a male mutant. At ten months of age, another female mutant was found to have tumorous tissue in the inguinal region. Tumorous tissue was observed at the last time point in two additional female mutants that were one year old. An example depicting the tumor in the pelvic region of one of these two mice is given in Fig. 4c. In the second female the tumor was located in the axilla. The hyper-intense area located in the spleen of one male, initially observed at the age of eight months, increased along the study. The development of this splenic tumor, which was histologically classified as hemangioma, and an example of the growth of the mammary tumor are depicted in Fig. 4. Correlations of mammary carcinomas observed by MRI and the corresponding histologic results are provided in the online supplemental data. Supplementary Fig. 3 tracks tumor progression over a two-month period, and Supplementary Fig. 4 presents examples of inguinal and axillary mammary carcinomas. In summary, two mutant females were sacrificed due to reaching humane endpoint and one mutant male was euthanized due to welfare reasons. Among the seven mutants per sex, cancerous tissue was detected on MRI in six females and one male. Detailed immunohistochemical results of tumor tissue samples found in this novel MVD013 mouse line are reported in the accompanying paper^11^.

**Table 1:** Overview of tumors detected during MRI in-vivo monitoring and tumor volumes derived.

Sex	Tumor type	Tumor location	Tumor volume (mm^3^)			
			6 months	8 months	10 months	12 months
f	Leukemia type	Spleen	1547^†^			
f	Mammary tumor	Axillar	*n. v*	1493^†^		
f	Mammary tumor	Inguinal	*n. v*	0.5	97	259
f	Mammary tumor	Axillar	*n. v*	*n. v*	*n. v*	800
f	Mammary tumor	Inguinal	*n. v*	*n. v*	4	16
f	Mammary tumor	Inguinal	*n. v*	*n. v*	*n. v*	1.5
m	Hemangioma	Spleen	*n. v*	2	7	30
m	–	–	*n. v*	*n. v.* ^†^		

A male mutant without imaging findings was sacrificed because it had reached the humane endpoint (*n. v.* not visible; ^†^end point).

### Relaxometry

#### T1 values vertebral bone marrow

Compared with controls, a steady decrease in the averaged relaxation time with age is observed in the male mutant groups. Substantially decreased T1 is observed in mutant females at twelve months, and in mutant males a significant decrease (*p* < 0.05) is observed at twelve months of age (Fig. 1i,j, see Supplementary Table 3 online). The stability of the serial experiments was assessed by analyzing the T1 times of an internal reference acquired in each of seven control and mutant mice. With respect to the corresponding mean T1 time, averaged over all measured time points, a variation of T1 between the different points of about ± 2% for the controls and ± 3% for the mutants was found. The detailed data of the internal reference is given in Supplementary Table 4 online. In comparison, a coefficient of variance (CoV) for daily repeatability of 2.34% was determined in a multisite reproducibility study on phantoms^23^.

#### T2 values spleen

The time course of the determined T2 times is shown in Fig. 1k,l. The data of the resulting splenic relaxation times are summarized in the Supplementary Table 3 online. In general, a faster T2 decay was revealed in female splenic tissue. Among controls, this sex difference proved statistically significant at six (p < 0.001) and twelve (*p* < 0.001) months of age. Female mutants were found to have statistically shorter T2 times at four (*p* < 0.05) and ten (*p* < 0.05) months of age. However, no genotype dependent alteration of the splenic T2 time was detected in the longitudinal study. In addition, no age-related changes in T2 times were observed for each individual group. To assess the stability of the relaxometry, the T2 value of the paravertebral muscle was analyzed. In terms of mean T2 relaxation, averaged over all measured time points, T2 varied by approximately ± 4% in both genotypes (see Supplementary Table 4 online). In comparison, for human brain imaging on clinical MRI systems, Gracien et al. reported intra-scanner reproducibility of 2.1%^24^.

### Histological analysis of lumbar vertebral bone marrow

**Figure 5:**
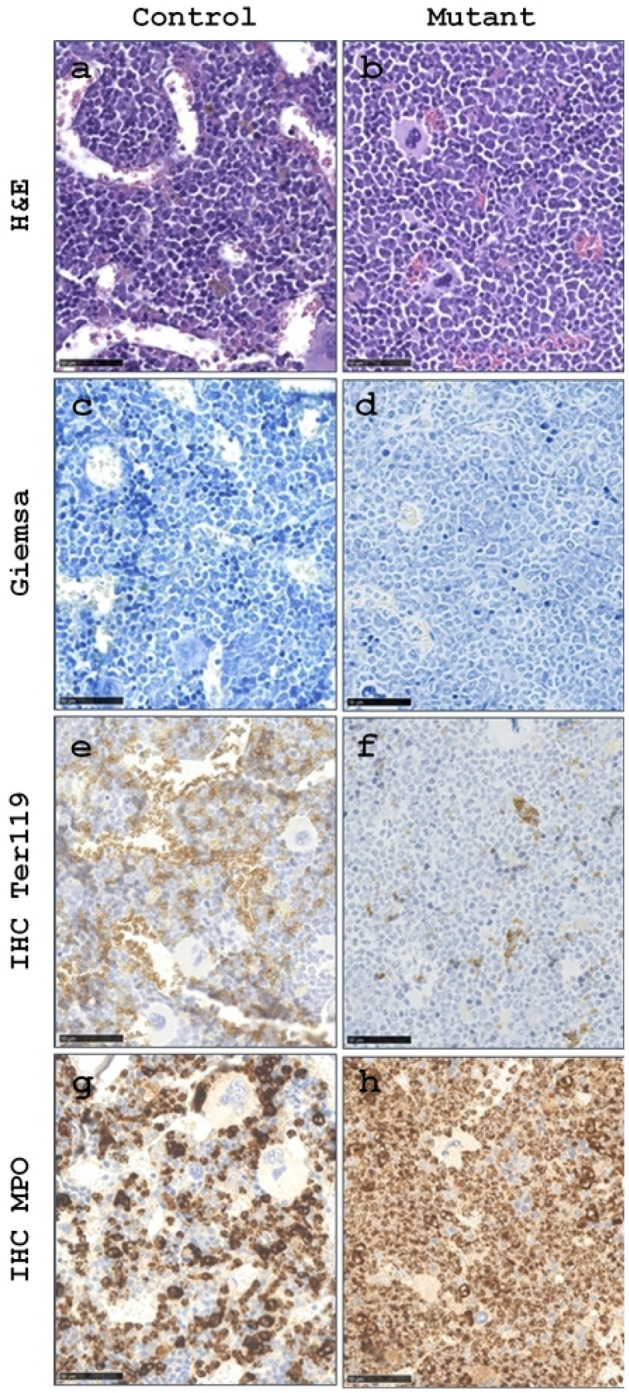
Representative images of bone marrow of the lumbar vertebrae from control (**a**, **c**, **e**, **g**) and mutant (**b**, **d**, **f**, **h**) mice. H&E and Giemsa stained sections (**a**–**d**) show an increase in hematopoietic cell density with a corresponding decrease in vascularization in the mutant compared to the control. Immunohistochemistry (IHC) using the Ter119 marker for mature and immature erythrocytes reveals the expected proportion of the erythrocyte to myeloid cells (**E**: M) ratio in the control animal (**e**), in contrast, a decreased amount of erythrocytes was detected in mutant (**f**). Whereas the IHC using the MPO marker demonstrates an increase in the number of myeloid cells, a shift to (**M**: E) ratio, in the mutant (**h**) compared to the control shown in (**g**).

To identify underlying changes in the vertebral bone marrow responsible for the shortening of T1 times in the mutants, we examined vertebral bone marrow sections from 12-month-old animals (3 per sex and genotype). The microscopic appearance indicates increased cellularity and a corresponding decrease in vascularization of the bone marrow in the mutant animals, detected by H&E and Giemsa. Interestingly, in contrast to the bone marrow composition of the femur, only scattered fat cells were detected in the vertebral bone marrow, regardless of genotype. Altered cellular composition with a decreased proportion of erythroid linage (E lineage) and an increased proportion of the myeloid lineage (M lineage) was detected by IHC staining. Therefore we diagnose a shift of hematopoietic cell lines from an (**E**:M) to a (**M**:E) ratio in the mutant animals compared to controls as depicted in Fig. 5. The alterations in cell composition were more pronounced in female animals. These results are consistent with previous observations in the spleen and femoral bone marrow^11^.

### Splenic iron load

**Figure 6:**
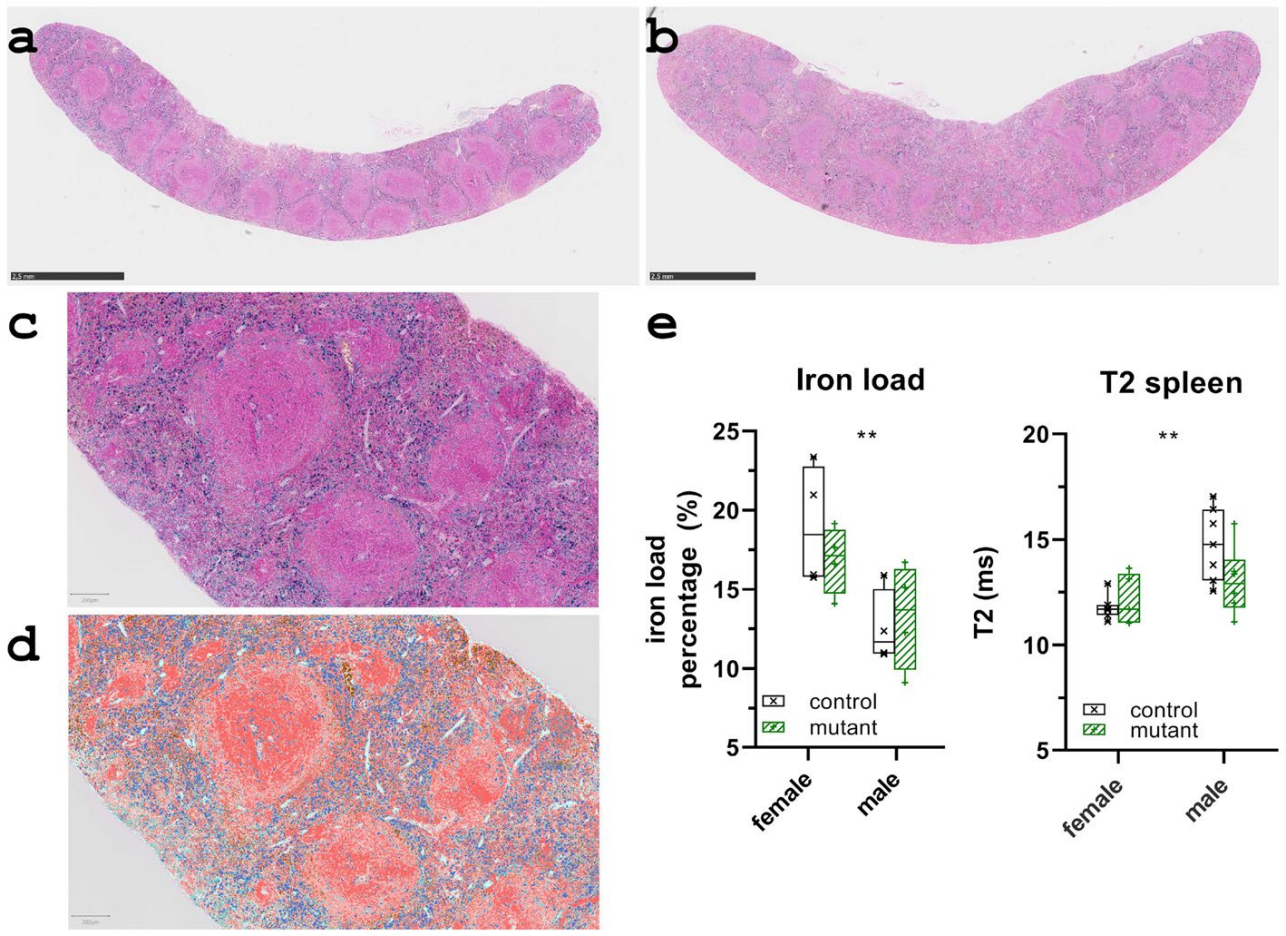
Quantification of the splenic iron load. (**a**) Example of a control spleen tissue and (**b**) of a mutant spleen tissue stained with Prussian blue. (**c**) The training region within a male control originally used for manual annotation with clearly identifiable, positively stained areas. (**d**) An example of the achieved performance of the trained pixel classifier, iron-loaded areas highlighted in dark blue. (**e**) Results of quantitative image analysis of spleen samples (females: 4/4, males: 4/4; age: 12 month) stained with Prussian blue confirmed the unchanged splenic iron load in C3H *Kit*^N824K/WT^ mice and the general sex-specific iron status. Consistently, shorter T2 times were measured in the spleen of females compared with males, both at 12 months of age (**, *p* ≤ 0.01).

An example of the staining results is shown in Fig. 6a for a male control and in Fig. 6b for a male mutant. The classifier used in the analysis of all samples was trained under supervision in a region of a male control shown in Fig. 6c. The result of the subsequent automatic classification applied to the training region is shown in Fig. 6d, with iron-containing areas highlighted in dark blue. No genotype related difference was observed between the eight controls and the eight mutants. Regardless of genotype, females had increased splenic iron load (*p* < 0.01), consistent with estimated shorter T2 values (*p* < 0.01) in females compared with males (Fig. 6e). An average iron load of 18.0 ± 3.1% and of 12.9 ± 2.7% was estimated for the female and the male samples, respectively (Fig. 6e). This sex specific difference is consistent with previous findings by other authors^25^.

## Discussion

In the present work, a longitudinal MRI study was performed in an activating *Kit* mutation bearing mouse model. This mutation is homologous to a variant identified in human patients suffering from GIST, germinomas, mastocytosis or AML^1^. In this follow up study, the temporal dynamics of the disease cascade manifested in different phenotypes including tumor development were characterized.

Body weight was measured before each imaging session. In the C3H control group, the observed sex differences in body weight were due to sexual dimorphism in the rate of weight gain, with females gaining less weight during the first six months^26^. In general, mouse strain differences affect the response to diet manipulation^27^. In the female group, the body weight of the mutants was significantly reduced from the age of eight months, probably due to the progression of the mammary tumors (8 months: *p* < 0.05; 10 months: *p* < 0.05; 12 months: *p* < 0.01).

The high-resolution 3D FISP data were used for MRI volumetry because this protocol resulted in a better delineation of the spleen. Compared with previously reported volumes, e.g. by Masi et al.^28^, derived splenic volumes in this study are larger. The larger volumes reported in the current study may be due to different strains. Sexual dimorphism of spleen volume was observed independent of genotype, with female mice exhibiting greater spleen volume compared with males. A similar finding was observed by Aguilar-Pimentel et al.^29^, who found increased spleen weight in females compared with males in C576BL/6 mice. A dramatic increase in volume was observed in three female mutants between the last two time points. In these mice, MRI detected mammary carcinomas for the first time at eight, ten, and twelve months of age, respectively. This association demonstrates the potential power of splenic MRI volumetry as a surrogate marker of disease status in this animal model. In a recent study using hepatocellular carcinoma as a model, Wei Jiang et al. demonstrated a positive correlation between spleen weight and tumor weight in tumor-bearing mice. In addition, based on a flow cytometry analysis of peripheral blood samples, these authors suggest that spleen weight may be a predictor of tumor prognosis^30^. The minireview by Laplane et al. discusses the main approaches of the conceptual views of cancer, the tumor-centric view, the tumor microenvironment (TME) view, and the “tumor organismal environment” (TOE) view^31^. Our observation of an enlarged spleen in a proportion of female C3H *Kit*^N824K/WT^ mice bearing mammary carcinoma is consistent with the concept of TOE and the view that cancer is a systemic disease^32^. The motivation of the current study was to characterize disease progression in a novel tumor model by in vivo imaging; a detailed treatise of breast carcinoma and its TOE is far beyond the scope of this study, the interested reader is referred to e.g. the review and references therein^31^.

GI organs were isotropically imaged using two protocols at a spatial resolution on the order of 250 µm^3^. Comparing the FISP and the fs-RARE data, the delineation of the GI organs is inferior on the FISP images, as exemplified in the Supplementary Fig. 5c,d. However, the content of the cecum appeared hypo-intense in both data. As a result, the cecal wall is clearly visible so that swellings and protruding structures can be identified. In case of the stomach, peristalsis prevented reliable analysis of stomach size or wall thickness. Lui et al. proposed a protocol using deuterated water, gadopentic acid, and a transient intestinal motility-inhibiting drug to evaluate gastric dysplasias and tumors, we observed an age-dependent cecal phenotype in our mouse model even without additional agents using the present protocol^33^. This phenotype ranged from a malformed small cecum in young mice to an enlarged obstipated cecum in older mutant mice. Previous histopathologic studies in other GIST models and in the present model have noted hyperproliferation of stromal cells in the wall of stomach and cecum (^10^,^11^). Accordingly, in the current MRI study, a striking thickening of the cecal wall was observed in the mutant cohort, likely reflecting hyperplasia of stromal cells in the GI wall (Fig. 3e,f, Supplementary Fig. 2). Alternatively, to image the immune response, advanced molecular imaging strategies such as bioluminescence could be used^34^. However, enlarged Peyer's patches in the cecum were clearly visible on the MRI data obtained. An increased incidence of these lymphoid nodules was observed in the mutants, as well as mammary tumors were observed in the majority of mutant females. It is noteworthy that as early as the middle of the last century, a direct correlation between the number of lymphoid aggregates and the occurrence of mammary tumors was found in mice^35^.

In addition to morphological examinations of GI organs, MRI relaxometry studies of haemopoietic tissues were performed. Bosbach et al.^6^ have shown the dysregulating impact of an activating *Kit* mutation on hematopoiesis. Such a hematologic phenotype was confirmed by Klein-Rodewald et al.^11^ for this novel *Kit*^N824K/WT^ model^11^, so a change in the T1 relaxation time of the vertebral bone marrow was expected. At twelve months of age, male mutants were found to have an altered T1 that was significantly shorter (*p* < 0.01) compared to controls. In the female mutants, a substantially shortened (*p* = 0.07) T1 was observed at twelve months of age (Fig. 1i,j, see Supplementary Table 3 online). Histologic analysis of vertebral bone marrow revealed increased cellularity and altered cell composition, consistent with analysis of femoral bone marrow that revealed increased numbers of hematopoietic cells in one-year-old C3H *Kit*^N824K/WT^ mutants^11^. In addition, the vascularization of the vertebral bone marrow appeared to be reduced in the mutant mice. Patient studies have shown that red bone marrow composition, particularly the relative amount of water-rich hematopoietic tissue compared with adipose tissue, affects T1 relaxation time^36,37^. In addition, an increase in iron stores impairs longitudinal relaxation, such as in the situation of iron overload and thalassemia^38^. In patients, polycythemia vera often develops into secondary myelofibrosis^39^. Sale et al. reported differential signal intensities on T1-weighted MRI images associated with secondary myelofibrosis^40^. However, histological analysis of the bone marrow of our mice revealed no obvious differences in the proportion of fat cells or signs of fibrosis in the mutant animals. As previously described for bone marrow in the sternum of mice, fat cells were almost absent^41^. Therefore, increased cellularity of the bone marrow with reduced interstitial fluid and vascularization could be the cause of the decreased T1 relaxation times in the mutants.

In the preceding study, increased red blood cell count, hematocrit, and hemoglobin were measured in peripheral blood samples from the novel model^11^. In addition, an altered cellular composition of the spleen was observed in *Kit*^N824K/WT^ animals. Consequently, an increased iron load in the spleen was expected in the mutants, which motivated the study of the transverse relaxation time T2. However, no statistical difference in splenic T2 was observed between genotypes and no age dependence was observed for T2. Accordingly, no genotypic change in the amount of ferric (Fe^3+^) iron ions was detectable in the spleen tissue samples stained with Prussian blue (Fig. 6a,b). Shorter T2 times were found in females, indicating a higher iron load. This difference was confirmed by analysis of tissue stained with Prussian blue, in which the visible Fe^3+^ pigmentation was more pronounced in females than in males (Fig. 6e). Thus, MRI relaxometry proved sufficiently sensitive to detect sex-specific small iron differences but did not reveal genotype-related differences in iron accumulation in the spleen of this mouse model^17,18,42,43^.

The presented MR study has limitations. It should demonstrate that a variety of disease-related phenotypes can be accurately monitored in vivo using MRI technology. The number of animals used in the current study may not be sufficient to draw definite conclusions about some aspects of the observed phenotypes. In particular, estimating the frequency of different tumor types and the variety of age of onset or tumor growth rates would require additional studies. Due to technical problems, T1 relaxometry measurements of a male control at the initial time point and a female mutant at the final time point were not performed. From three animals of each group, the T2 data of the first measurement had to be excluded due to an inconsistent setting of the MSME protocol. At the third time point, T1 and T2 data from a male mutant were confounded by movement and were therefore excluded. The resulting analyzed animals at each observation point are listed in the footnote to the relaxometry results in Supplementary Table 3. Assuming that GISTs would develop, the study design focused on imaging of the abdominal organs. Consequently, the standard field of view (FOV) setting did not fully cover the anatomical region of the chest. Therefore, in two mice that had mammary carcinoma in the axilla, the volume estimate may have underestimated the true tumor size. For future studies, the FOV should be enlarged to cover the chest, axillary, and neck regions as well.

MRI examinations revealed tumorous tissue in the C3H *Kit*^N824K/WT^ mutant animals. The first mammary tumors were detected at the age of eight months, with the smallest tumor being less than one cubic millimeter in size. In addition, a leukemic type and a hemangioma were observed in the spleens of a female and male mutant, respectively. Volumetric analysis and characterization of individual progression profiles were successfully performed. Cancer detection was based on the fs-RARE technique, using an imaging protocol similar to that used, for example, by Fan et al. to study mammary cancer progression in mice^44^. For future studies, it is planned to additionally monitor tumor metabolism, for example by chemical exchange saturation transfer (CEST) or magnetic resonance spectroscopy (MRS)^45–47^. In summary, a longitudinal study characterizing for the first time the progression of different phenotypes in a novel cancer model was performed. It has been shown that the proposed MRI protocols can monitor tumor development and important physiological changes that are not accessible at early stages by external physical observations. A premise believed to help elucidate the complex interaction of genetic background on disease severity and progression in this novel C3H *Kit*^N824K/WT^ and other *Kit*-induced cancer models. The authors are confident that the presented in vivo MRI characterization of the time-dependent phenotypes seen in this novel disease model will make a valuable contribution to gastrointestinal and mammary cancer research.

## Methods

### Animals

For the purpose of this study C3H *Kit*^N824K/WT^ heterozygous mutant and wildtype littermates, seven animals per sex and genotype, in total twenty-eight animals were used. Details on the model generation can be found in^48^. The mice were housed in individually ventilated cages in a temperature-controlled room at 22 °C under 12 h light/dark cycle with free access to bottled water and a standard diet. On the day of the imaging experiments mice were fasted six hours prior measurements in order to reduce peristalsis^49^. After the last time point, mice were sacrificed for the histopathological analysis, as well as mice which had to be euthanized earlier due to reaching humane end point. All studies reported here were conducted in accordance with the principle of the “Three Rs” (EU Regulatory Framework Directive 2010/63/EU) and in accordance with the applicable animal welfare and other regulations in Germany. The tests were approved by the responsible authority of the district government of Upper Bavaria “Regierung von Oberbayern, Sachgebiet 54 – Tierschutz; München, Germay” (registration no. ROB-55.2–2532.Vet_02-16–46) and carried out in compliance with the ARRIVE guidelines.

### MR imaging

The MRI experiments were performed on a 9.4 T Biospec 94/20 USR (Bruker BioSpin GmbH, Germany) small animal system equipped with a 675 mT/m gradient system B-GA12S HP (Bruker BioSpin GmbH, Germany). For signal acquisition a quadrature transmit/receive coil with an inner diameter of 35 mm (M2M imaging corporation, USA) was used. Imaging experiments were performed every other month starting at an age of two months until twelve months. Relaxometry data was acquired according to this scheme starting at the age of four months. The mice were anesthetized by isoflurane inhalation, initiated with 5% and then sustained with 1–3% isoflurane in 100% oxygen. Anesthetized animals were placed in supine position and covered by a warming cover, which was connected to a heating circuit. A small animal physiological monitoring system (SA Instruments, USA) was used to monitor respiration rate by a pressure sensitive pad and body temperature by a rectal thermometer. Prior to imaging body weight of each individual animal was recorded.

#### MR morphometry

The protocol for the examination of the gastrointestinal organs comprised a two-dimensional fat-suppressed multi-slice RARE sequence (2D fs-RARE) and a three-dimensional FISP sequence (3D FISP), with each respiratory gating applied. The measurement parameters of the 2D fs-RARE and the 3D FISP sequence are summarized in Table 2. For volumetric analysis the segmentation of the spleen and the cecum was carried out in semi-automatic fashion using the program ITK-SNAP^50^. Cecal tissue was delineated in the 2D fs-RARE data while splenic tissue was identified on the 3D FISP images.

**Table 2:** Overview of the applied MR protocols.

Protocol	TR (ms)	TE (ms)	Flip angle	Rare factor	Bandwidth (kHz)	Averages	FOV (mm^2^)	Spatial resolution (mm^3^)	
**MR morphometry**									
*2D RARE*	2475	12.6	90°/180°	4	81.5	4	35 × 38	0.18 × 0.19 × 0.25	
*3D FISP*	5	1.4	15°	–	119.05	4	35 × 38	0.22 × 0.24 × 0.25	

Protocol	TR (ms)	TE (ms)	Flip angle	Images	TI (ms)	Bandwidth (kHz)	Averages	FOV (mm^2^)	Spatial resolution (mm^3^)
**MR relaxometry**									
*T1: IR-FISP*	10,500	2.65	180°/10°	7	57/Δ480	62.5	2	30 × 30	0.19 × 0.19 × 1
*T2: MSME*	2000	6	90°/180°	14	–	98.7	1	30 × 30	0.19 × 0.19 × 0.75

The MR morphometry comprised a high resolution 2D rapid acquisition with relaxation enhancement (RARE) and a 3D fast imaging with steady-state precession (FISP) sequence. The T1 relaxation time was measured based on a Look-Locker approach sampling the T1 dynamic using an inversion recovery FISP (IR-FISP) technique. The transversal relaxation time T2 was derived from a multi-slice multi-echo (MSME) protocol.

#### MR relaxometry

The longitudinal relaxation time T1 of vertebral bone marrow was measured with a single slice inversion recovery FISP (IR-FISP) Look-Locker technique^51^. The single slice was orientated so as to cover the lumbosacral vertebral column in coronal slice orientation; details of the IR-FISP protocol are given in Table 2. Respiration triggering was applied to initialize the acquisition train, with an acquisition delay of 100 ms after the respiration trigger. During acquisition mice were kept at nearly constant breathing rate of 40 cycles/min. The obtained signal time course can be described by

A $$S\left( {TI} \right) = a - b \times e^{{ - TI/T1^{*} }} \quad T1 = T1^{*} \times \left( {\frac{b}{a} - 1} \right)$$

where *TI* is the time following the inversion pulse, and T1^*^ is the apparent *T1* under the influence of Look-Locker saturation^52^. For each mouse one region of interest (ROI) within the bone marrow of the third lumbar vertebra, one ROI within the paravertebral muscle, and one in the background noise were manually delineated (Supplementary Fig. 5a). In case an image was corrupted with respiration motion it was manually excluded from fitting. The longitudinal relaxation times T1 were determined by model fitting, based on Equations A, to the mean signal intensity of each ROI. The transversal relaxation time T2 was determined by a respiratory triggered multi-slice multi-echo spin echo sequence (T2-MSME), parameters are given in Table 2. For each mouse one ROI within the spleen, one ROI within the paravertebral muscle, and one in the background noise were manually delineated (Supplementary Fig. 5b). The transversal relaxation time T2 was estimated based on a mono-exponential fitting model of the transverse signal decay

B $$S\left( {TE} \right) = M0 \times e^{ - TE/T2} + c$$

where *M0* is the initial signal intensity, *TE* is the echo time, and *c* is a fitted offset to account for image noise. The transversal relaxation times T2 were determined by model fitting, based on Equation B, to the mean signal intensity of each ROI. Prior computation of the T2 the first echo was excluded from the MSME data^53^. The estimation of the T1 and T2 times was performed in iterative reweighted least squares routine using scientific computing software Matlab (2019b, The Mathworks, Inc., Natick, USA).

### Histopathological analysis

#### Macroscopic and histological analysis

At the end of the study, at twelve months of age, the mice were euthanized and necropsied. The weight of the visceral organs was determined and the macroscopic findings were documented by photographs. Inner organs and tumor tissues were fixed in 4% buffered formalin, embedded in paraffin and the tissue sections (3 µm) were stained with hematoxylin and eosin. Bone marrow of the lumbar vertebrae was processed through conventionally fixed sections of decalcified bone embedded in paraffin^54^. The evaluation of H&E- and Giemsa-stained tissue sections served as the screening test for bone marrow histopathology. This assessment included an estimate of cellular density. The term ‘bone marrow cellular density’ refers to the amount or percentage of hematopoietic cells relative to supportive tissue composed of marrow fat, interstitial vascularization and mesenchymal stem cells. In addition, the ratio of erythroid to myeloid cells (E: M) was determined. Cells of hematopoietic lineage are difficult to distinguish from the many other nucleated cells in H&E- and Giemsa-stained bone marrow. Therefore, we used immunohistochemical markers for the different lines: (1) TER-119 which is a marker for mature and immature erythrocytes and in addition, (2) myeloperoxidase (MPO), which is a peroxidase enzyme that is most abundantly expressed in granulocytes of the myeloid lineage as described in the accompanying paper^11^. Macroscopic and histological analysis was performed by experienced mouse pathologists.

#### Iron deposits histomorphometry

In order to correlate the transversal relaxation time T2 obtained by MR relaxometry with splenic iron (Fe^3+^) load Prussian blue staining of splenic tissue samples was performed^55^. For each genotype, four female and four male samples were processed. Samples were collected after the last imaging session at twelve months of age. Bioimage analysis software QuPath was used for the quantification^56^. Manually guided training was applied in order to tune the automated pixel classifier. This training phase utilized the manual annotation of variably stained areas and supervised the outcome of the automated classifier in an iterative fashion^43^. Positively stained regions were identified as iron loaded areas, negatively stained regions were combined in a notional category. For each tissue section, the iron load was expressed as the percentage of the positive areas relative to the total areas detected.

### Statistical analysis

GraphPad Prism version 8.4.3 (GraphPad Software, La Jolla California USA) was used for all statistical analyses. Data were tested for normality using the Kolmogorov–Smirnov method. The comparison of two normally distributed variables was performed using a unpaired two-tailed t test. Variables that did not conform to a normal distribution were compared using two-tailed Mann–Whitney test. Results with *p* value ≤ 0.05 were considered statistically significant (*, *p* ≤ 0.05; **, *p* ≤ 0.01; ***, *p* ≤ 0.001). Data at each observation time point are presented as mean ± standard deviation across the corresponding group.

## Supplementary Information

Supplementary Information.

## Data Availability

Data will be made available from the co-author H. Fuchs on reasonable request.

## References

[1] Abbaspour Babaei M, Kamalidehghan B, Saleem M, Huri HZ, Ahmadipour F (2016). Receptor tyrosine kinase (c-Kit) inhibitors: A potential therapeutic target in cancer cells. Drug Des. Dev. Ther..

[2] Lennartsson J, Ronnstrand L (2006). The stem cell factor receptor/c-Kit as a drug target in cancer. Curr. Cancer Drug Targets.

[3] Gunawan B (2008). Knock-in murine models of familial gastrointestinal stromal tumours. J. Pathol..

[4] Pham DDM, Guhan S, Tsao H (2020). KIT and melanoma: Biological insights and clinical implications. Yonsei Med. J..

[5] Ke H, Kazi JU, Zhao H, Sun J (2016). Germline mutations of KIT in gastrointestinal stromal tumor (GIST) and mastocytosis. Cell Biosci..

[6] Bosbach B (2012). Imatinib resistance and microcytic erythrocytosis in a KitV558Delta;T669I/+ gatekeeper-mutant mouse model of gastrointestinal stromal tumor. Proc. Natl. Acad. Sci. U S A.

[7] Gerbaulet A (2011). Mast cell hyperplasia, B-cell malignancy, and intestinal inflammation in mice with conditional expression of a constitutively active kit. Blood.

[8] Nakai N (2008). A mouse model of a human multiple GIST family with KIT-Asp820Tyr mutation generated by a knock-in strategy. J. Pathol..

[9] Rubin BP (2005). A knock-in mouse model of gastrointestinal stromal tumor harboring kit K641E. Cancer Res..

[10] Sommer G (2003). Gastrointestinal stromal tumors in a mouse model by targeted mutation of the Kit receptor tyrosine kinase. Proc. Natl. Acad. Sci. U S A.

[11] Klein-Rodewald, T. *et al.* New C3H KitN824K/WT cancer mouse model develops late-onset malignant mammary tumors with high penetrance. Preprint at https://www.biorxiv.org/content/10.1101/2022.08.23.504938v1, 10.1101/2022.08.23.504938. 10.1038/s41598-022-23218-5 PMC9671887 36396684

[12] Jansen SA (2009). Magnetic resonance imaging of the natural history of in situ mammary neoplasia in transgenic mice: A pilot study. Breast Cancer Res..

[13] Garbow JR, Wang M, Wang Y, Lubet RA, You M (2008). Quantitative monitoring of adenocarcinoma development in rodents by magnetic resonance imaging. Clin. Cancer Res..

[14] Casali PG (2018). Gastrointestinal stromal tumours: ESMO-EURACAN Clinical Practice Guidelines for diagnosis, treatment and follow-up. Ann. Oncol..

[15] Hensley H (2007). Magnetic resonance imaging for detection and determination of tumor volume in a genetically engineered mouse model of ovarian cancer. Cancer Biol. Ther..

[16] Dimitrakopoulou-Strauss A (2017). Imaging therapy response of gastrointestinal stromal tumors (GIST) with FDG PET, CT and MRI: A systematic review. Clin. Transl. Imaging.

[17] Hitti E (2010). MRI quantification of splenic iron concentration in mouse. J. Magn. Reson. Imaging.

[18] Jackson LH (2017). Non-invasive MRI biomarkers for the early assessment of iron overload in a humanized mouse model of beta-thalassemia. Sci. Rep..

[19] Jensen KE (1990). Localized in vivo proton spectroscopy of the bone marrow in patients with leukemia. Magn. Reson. Imaging.

[20] Lecouvet FE (1998). Chronic lymphocytic leukemia: Changes in bone marrow composition and distribution assessed with quantitative MRI. J. Magn. Reson. Imaging.

[21] Schick F (1993). Leukemic red bone marrow changes assessed by magnetic resonance imaging and localized 1H spectroscopy. Ann. Hematol..

[22] Newberry RD, Lorenz RG (2005). Organizing a mucosal defense. Immunol. Rev..

[23] Waterton JC (2019). Repeatability and reproducibility of longitudinal relaxation rate in 12 small-animal MRI systems. Magn. Reson. Imaging.

[24] Gracien RM (2020). How stable is quantitative MRI? Assessment of intra- and inter-scanner-model reproducibility using identical acquisition sequences and data analysis programs. Neuroimage.

[25] Ward, J. M., Mann, P. C., Morishima, H. & Frith, C. H. in *Pathology of the mouse* (ed R. R. Maronpot) 333–337 (Cache River Press, 1999).

[26] Casimiro I, Stull ND, Tersey SA, Mirmira RG (2021). Phenotypic sexual dimorphism in response to dietary fat manipulation in C57BL/6J mice. J. Diabetes Complicat..

[27] Kanasaki K, Koya D (2011). Biology of obesity: Lessons from animal models of obesity. J. Biomed. Biotechnol..

[28] Masi B (2015). In vivo MRI assessment of hepatic and splenic disease in a murine model of schistosomiasis [corrected]. PLoS Negl. Trop. Dis..

[29] Aguilar-Pimentel JA (2020). Increased estrogen to androgen ratio enhances immunoglobulin levels and impairs B cell function in male mice. Sci. Rep..

[30] Jiang W, Li Y, Zhang S, Kong G, Li Z (2021). Association between cellular immune response and spleen weight in mice with hepatocellular carcinoma. Oncol. Lett..

[31] Laplane L, Duluc D, Bikfalvi A, Larmonier N, Pradeu T (2019). Beyond the tumour microenvironment. Int. J. Cancer.

[32] McAllister SS, Weinberg RA (2014). The tumour-induced systemic environment as a critical regulator of cancer progression and metastasis. Nat. Cell Biol..

[33] Berr SS, Roche JK, El-Rifai W, Smith MF, Powell SM (2003). Magnetic resonance imaging of gastric cancer in Tff1 knock-out mice. Magn. Reson. Med..

[34] Gross S, Moss BL, Piwnica-Worms D (2007). Veni, vidi, vici: In vivo molecular imaging of immune response. Immunity.

[35] Kelsall MA (1946). Number of Peyer's patches in mice belonging to high and low mammary tumor strains. Proc. Soc. Exp. Biol. Med..

[36] Floyd RA, Yoshida T, Leigh JS (1975). Changes of tissue water proton relaxation rates during early phases of chemical carcinogenesis. Proc. Natl. Acad. Sci. U S A.

[37] Shah S, Ranade SS, Kasturi SR, Phadke RS, Advani SH (1982). Distinction between normal and leukemic bone marrow by water protons nuclear magnetic resonance relaxation times. Magn. Reson. Imaging.

[38] Cuthbert D, Stein BL (2019). Polycythemia vera-associated complications: pathogenesis, clinical manifestations, and effects on outcomes. J. Blood Med..

[39] Barraco D (2017). Prognostic impact of bone marrow fibrosis in polycythemia vera: Validation of the IWG-MRT study and additional observations. Blood Cancer J..

[40] Sale GE, Deeg HJ, Porter BA (2006). Regression of myelofibrosis and osteosclerosis following hematopoietic cell transplantation assessed by magnetic resonance imaging and histologic grading. Biol. Blood Marrow Transplant..

[41] Meza-Leon B (2021). Human, mouse, and dog bone marrow show similar mesenchymal stromal cells within a distinctive microenvironment. Exp. Hematol..

[42] Hocq A (2015). Effect of magnetic field and iron content on NMR proton relaxation of liver, spleen and brain tissues. Contrast Media Mol. Imaging.

[43] Cesta MF (2006). Normal structure, function, and histology of the spleen. Toxicol. Pathol..

[44] Fan X (2014). Mammary cancer initiation and progression studied with magnetic resonance imaging. Breast Cancer Res..

[45] van Zijl PCM (2021). Hyperpolarized MRI, functional MRI, MR spectroscopy and CEST to provide metabolic information in vivo. Curr. Opin. Chem. Biol..

[46] Fiordelisi MF, Cavaliere C, Auletta L, Basso L, Salvatore M (2019). Magnetic resonance imaging for translational research in oncology. J. Clin. Med..

[47] Zhou R (2018). Glutamate-weighted chemical exchange saturation transfer magnetic resonance imaging detects glutaminase inhibition in a mouse model of triple-negative breast cancer. Cancer Res..

[48] Aigner B (2011). Generation of N-ethyl-N-nitrosourea-induced mouse mutants with deviations in hematological parameters. Mamm. Genome.

[49] Grimm J, Potthast A, Wunder A, Moore A (2003). Magnetic resonance imaging of the pancreas and pancreatic tumors in a mouse orthotopic model of human cancer. Int. J. Cancer.

[50] Yushkevich PA (2006). User-guided 3D active contour segmentation of anatomical structures: Significantly improved efficiency and reliability. Neuroimage.

[51] Crawley AP, Henkelman RM (1988). A comparison of one-shot and recovery methods in T1 imaging. Magn. Reson. Med..

[52] Deichmann R, Haase A (1992). Quantification of T1 values by SNAPSHOT-FLASH NMR imaging. J. Magn. Reson..

[53] McPhee KC, Wilman AH (2018). Limitations of skipping echoes for exponential T2 fitting. J. Magn. Reson. Imaging.

[54] Elmore SA (2006). Enhanced histopathology of the bone marrow. Toxicol. Pathol..

[55] Perls M (1867). Nachweis von Eisenoxyd in gewissen Pigmenten. Virchows Arch. A Pathol. Anat. Histopathol..

[56] Bankhead P (2017). QuPath: Open source software for digital pathology image analysis. Sci. Rep..

