# Supplementary material for: Monitoring longitudinal disease progression in a novel murine Kit tumor model using high-field MRI
Source: Sci Rep. 2022 Aug 26;12:14608. doi: 10.1038/s41598-022-17880-y (PMC9418174; doi:10.1038/s41598-022-17880-y)
Supplement: Supplementary file 1 — Supplementary Information. [file 41598_2022_17880_MOESM1_ESM.pdf]

Monitoring longitudinal disease progression in a novel murine *Kit* tumor model using high-field MRI

Markus Kraiger [1*], Tanja Klein-Rodewald [1], Birgit Rathkolb [1,2,3], Julia Calzada-Wack [1], Adrián Sanz-Moreno [1], Helmut Fuchs [1], Eckhard Wolf [3], Valérie Gailus-Durner [1], Martin Hrabě de Angelis [1,2,4]

[1]Institute of Experimental Genetics, German Mouse Clinic, Helmholtz Zentrum München, German Research Center for Environmental Health, Neuherberg, Germany

[2]German Center for Diabetes Research, Helmholtz Zentrum München, German Research Center for Environmental Health, Neuherberg, Germany

[3]Institute of Molecular Animal Breeding and Biotechnology, Gene Center, Ludwig-Maximilians-University München, Munich, Germany

[4]Chair of Experimental Genetics, TUM School of Life Sciences, Technische Universität München, Freising, Germany

*** Correspondence:**
Corresponding Author: Markus Kraiger
markus.kraiger@helmholtz-muenchen.de

Keywords: *Kit* mutation, animal model, mammary cancer, gastrointestinal stromal tumor, magnetic resonance imaging

Abstract

Animal models are an indispensable platform used in various research disciplines, enabling, for example, studies of basic biological mechanisms, pathological processes and new therapeutic interventions. In this study, we applied magnetic resonance imaging (MRI) to characterize the clinical picture of a novel N-ethyl-N-nitrosourea-induced (ENU) *Kit*-mutant mouse in vivo. Seven C3H $Kit^{N824K/WT}$ mutant animals each of both sexes and their littermates were monitored every other month for a period of twelve months. MRI relaxometry data of hematopoietic bone marrow and splenic tissue as well as high-resolution images of the gastrointestinal organs were acquired. Compared with controls, the mutants showed a dynamic change in the shape and volume of the cecum and enlarged Peyer´s patches were identified throughout the entire study. Mammary tumors were observed in the majority of mutant females and were first detected at eight months of age. Using relaxation measurements, a substantial decrease in longitudinal relaxation times in hematopoietic tissue was detected in mutants at one year of age. In contrast, transverse relaxation time of splenic tissue showed no differences between genotypes, except in two mutant mice, one of which had leukemia and the other hemangioma. In this study, in vivo MRI was used for the first time to thoroughly characterize the evolution of systemic manifestations of a novel *Kit*-induced tumor model and to document the observable organ-specific disease cascade.

Supplementary Material

Supplementary Table 1

Supplementary Table 1: Results of the MR volumetry and the body weight data. The mutant mice show a time-varying morphological phenotype. Compared to control mice, the cecal volume is initially decreased, whereas it increases dramatically at later observation time points. Sex specific differences are observed in bodyweight (males are heavier than females) and spleen volume (females have a larger spleen). Unless otherwise stated differently, data of seven animals per group are shown at each time point (data represented as mean +/- standard deviation).

**male**	body weight (g)			splenic volume (mm³)			cecal volume (mm³)		
time	**control**	**mutant**	***p* value**	**control**	**mutant**	***p* value**	**control**	**mutant**	***p* value**
t1	27.54 ± 2.01	25.56 ± 1.85	0.1008	114.43 ± 13.48	119.20 ± 10.81	0.3829	189.95 ± 49.50	72.52 ± 37.09	**0.0002**
t2	33.20 ± 2.24	31.11 ± 1.69	0.0937	123.89 ± 37.73	117.07 ± 8.06	0.4009	239.61 ± 48.53	131.45 ± 41.65	**0.0014**
t3	36.87 ± 3.39	34.37 ± 1.87	0.1402	120.37 ± 29.90	114.69 ± 13.14	0.62	282.26 ± 48.40	166.80 ± 31.26	**0.0004**
t4	39.37 ± 3.73	37.39 ± 3.38	0.3532	127.39 ± 31.43	120.33 ± 11.71	0.8048	253.04 ± 93.42	302.60 ± 103.39	0.4557
t5	39.17 ± 3.56	36.95 ± 2.67$^{\Delta}$	0.2723	132.24 ± 37.82	132.55 ± 20.68$^{\Delta}$	0.6282	243.87 ± 75.31	374.37 ± 129.84$^{\Delta}$	0.071
t6	39.80 ± 3.82	36.48 ± 2.30$^{\Delta}$	0.1061	146.11 ± 37.13	131.50 ± 26.22$^{\Delta}$	0.4736	226.46 ± 57.44	466.43 ± 140.00$^{\Delta}$	**0.0029**

$^{\Delta}$… n = 6

**female**	body weight (g)			splenic volume (mm³)			cecal volume (mm³)		
time	**control**	**mutant**	***p* value**	**control**	**mutant**	***p* value**	**control**	**mutant**	***p* value**
t1	22.44 ± 0.86	21.69 ± 1.59	0.3238	128.61 ± 10.51	127.89 ± 17.48	0.9317	164.89 ± 50.38	62.74 ± 24.93	**0.0008**
t2	28.70 ± 1.64	27.70 ± 1.53	0.2963	137.74 ± 16.87	150.67 ± 24.25	0.3048	224.09 ± 27.28	132.18 ± 64.32	**0.0073**
t3	33.69 ± 1.36	30.29 ± 3.68	0.0551	135.61 ± 10.32	142.02 ± 13.83$^{\Delta}$	0.3992	201.37 ± 50.97	186.50 ± 74.75	0.6943
t4	38.17 ± 3.81	33.80 ± 2.07$^{\Delta}$	**0.0415**	140.20 ± 14.43	151.85 ± 20.70$^{\Delta}$	0.2972	131.33 ± 52.43	328.88 ± 119.75$^{\Delta}$	**0.004**
t5	37.96 ± 3.37	33.14 ± 2.61‡	**0.0349**	134.60 ± 13.63	143.72 ± 11.33‡	0.2899	189.79 ± 69.68	428.94 ± 103.42‡	**0.0014**
t6	38.81 ± 2.36	32.88 ± 2.18‡	**0.0023**	151.54 ± 15.65	187.14 ± 42.70‡	0.0945	178.32 ± 52.75	483.16 ± 157.92‡	**0.0015**

$^{\Delta}$… n = 6; ‡… n = 5

Supplementary Table 2

Supplementary Table 2: Results of the counting of Peyer´s patches. 2D fs-RARE volumetric data were analyzed to detect patches in the cecal lumen. A substantially increased number of patches was detected in the mutant cohort compared to controls. For each group, the numbers represent the sum of the patches counted at each time point. Unless otherwise stated differently, data of seven animals per group are shown at each time.

**male**	Peyer´s Patches Count		**female**	Peyer´s Patches Count	
time	**control**	**mutant**	time	**control**	**mutant**
t1	6	54	t1	8	58
t2	4	56	t2	4	55
t3	6	42	t3	3	46
t4	10	57	t4	14	38
t5	7	53 $^{\Delta}$	t5	5	30‡
t6	20	38 $^{\Delta}$	t6	16	28‡

$^{\Delta}$… n = 6; ‡… n = 5

Supplementary Figure 1

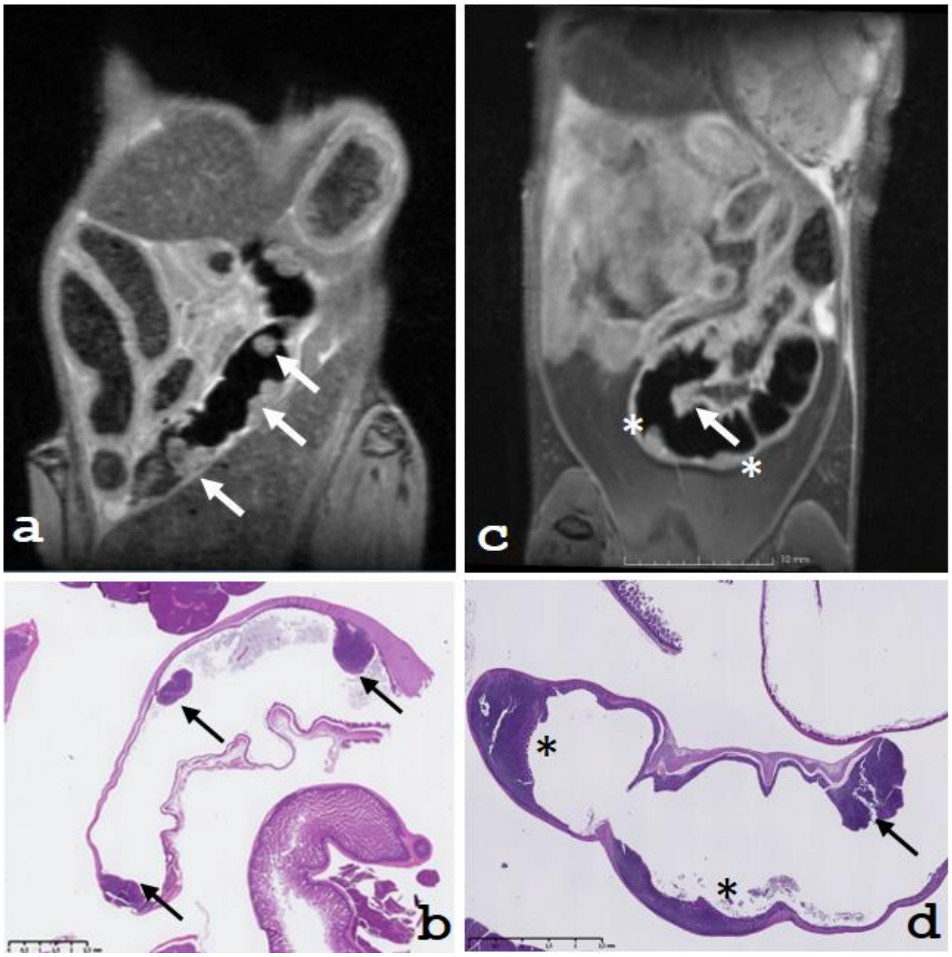

Supplementary Figure 1. Examples of the age dependent appearance of Peyer´s patches. Prominent patches (white arrows) observed by fs-RARE in the cecum of a six-month old female mutant **(a)**. Reaching humane endpoint, this six-month old mouse was examined histopathologically. Corresponding H&E stained tissue slide revealing enlarged patches (black arrows) **(b)**. Compressed patches (white stars) were observed at later time points, as a consequence of cecal constipation. An example of an eight-month old female mutant is given in **(c)**. Reaching humane endpoint, this eight-month old female was examined histopathologically. The compressed nodules (black stars) were also observed on the corresponding H&E stained tissue cut **(d)**. Scale bar = 2.5 mm.

Supplementary Figure 2

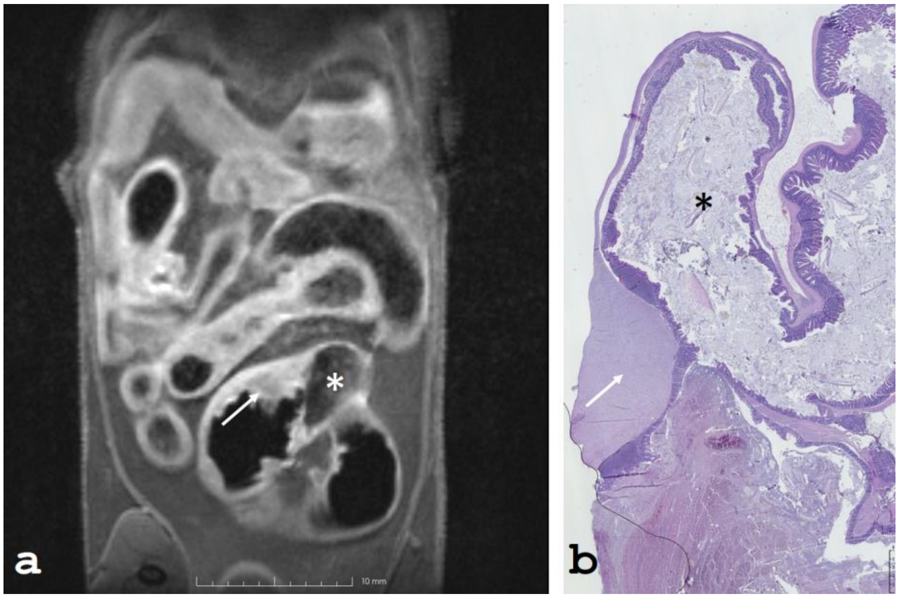

Supplementary Figure 2: MRI findings in comparison with histology of a one-year-old female mutant. Conspicuous cecal wall thickening (thickening indicated by white arrow) at the ileocecal junction, clearly visible in the 2D-fs RARE data **(a)**. Marginal MR signal originates from the lumen of the ileum (lumen marked by white asteriks). The corresponding H&E stained slide of the ileocecal junction confirmed thickening of the muscularis propria of the cecum caused by hyperplastic ICCs, indicated by a white arrow **(b)**. The lumen of the ileum is marked by a black asterisk. Scale bar = 1 mm.

Supplementary Figure 3

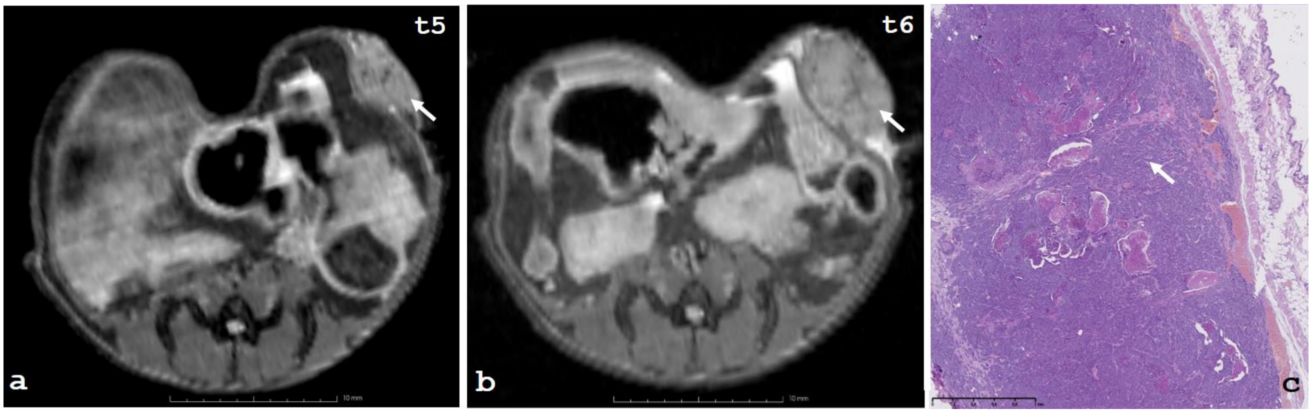

Supplementary Figure 3: Tracking mammary cancer development in a female mutant over a two-month period. Inguinal mammary tumor (indicated by a white arrow) seen on 2D fs-RARE axial data of a ten-month-old female mutant **(a)**. Analysis of the MRI data showed that the extent of the cancerous tissue was 97 mm^3. At twelve months of age, a tumor (white arrow) volume of 259 mm^3 was estimated by MRI in the same mouse **(b)**. The affected mammary gland is seen on the H&E-stained tissue section where subcutaneous fat and skin are to the right of the tumorous tissue at 12 months of age **(c)**. Scale bar = 1 mm.

Supplementary Figure 4

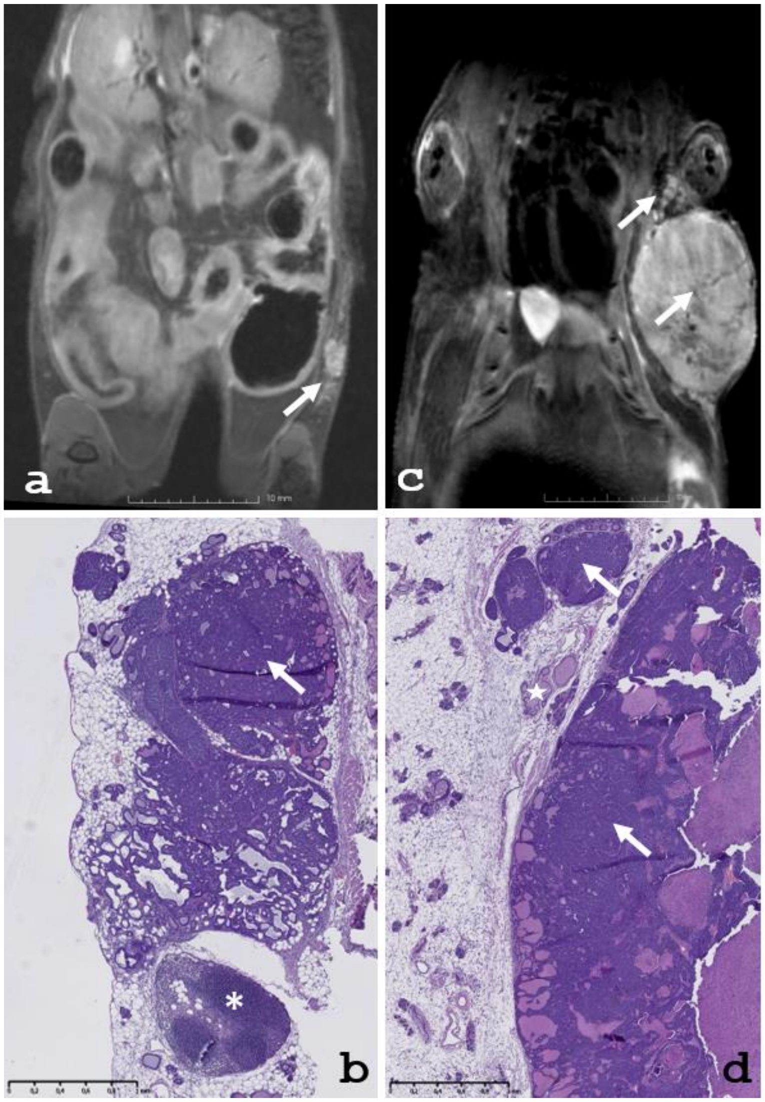

Supplementary Figure 4: Comparison of MRI findings with histology of a one-year-old female mutant. Tumorous tissue (indicated by white arrow) at the inguinal region, clearly visible in the 2D-fs RARE data **(a)**. The corresponding H&E-stained section revealed a smaller mammary carcinoma, indicated by a white arrow, close to the inguinal lymph node (marked by a white asterisk) **(b).** At the axillar region of this mouse, an additional tumor mass is observed on the 2D-fs RARE image (white arrows) **(c).** Mammary carcinoma is seen on the corresponding H&E-stained tissue section, indicated by white arrows. In addition, normal mammary gland ducts can be seen on this section, indicated by a white star **(d).** Scale bar = 1 mm.

Supplementary Table 3

Supplementary Table 3: Results of the MR relaxometry. At the last two observation points, a trend toward shortening of T1 was observed in both mutant groups. In the male mutants, a significant difference was observed at twelve months of age. The transversal decay time T2 was not significantly altered between genotypes; nevertheless, MR relaxometry revealed sex differences that were more pronounced in the control mice. Unless otherwise stated differently, data of seven animals per group are shown at each time point (data represented as mean +/- standard deviation).

**male**	T1 bone marrow (ms)			T2 splenic tissue (ms)		
time	**control**	**mutant**	***p* value**	**control**	**mutant**	***p* value**
t2	1604.84 ± 92	1624.08 ± 39$^{\Delta}$	0.6692	14.22 ± 0.55$^{\lozenge}$	13.49 ± 0.48$^{\lozenge}$	0.1143
t3	1627.98 ± 45	1579.82 ± 25	0.0530	14.59 ± 1.67	12.98 ± 1.47	0.1030
t4	1614.44 ± 54	1576.97 ± 33$^{\Delta}$	0.1996	14.50 ± 1.81	12.98 ± 1.25$^{\Delta}$	0.1391
t5	1629.16 ± 61	1549.93 ± 60$^{\Delta}$	0.0531	14.25 ± 1.17	12.96 ± 1.12$^{\Delta}$	0.0995
t6	1625.47 ± 38	1543.88 ± 47$^{\Delta}$	**0.0086**	14.78 ± 1.58	13.03 ± 1.46$^{\Delta}$	0.0854

$^{\Delta}$… n = 6, $^{\lozenge}$… n = 4

**female**	T1 bone marrow (ms)			T2 splenic tissue (ms)		
time	**control**	**mutant**	***p* value**	**control**	**mutant**	***p* value**
t2	1584.32 ± 39	1625.68 ± 44	0.1282	12.76 ± 1.16$^{\lozenge}$	11.82 ± 0.25$^{\lozenge}$	0.3429
t3	1585.84 ± 64	1624.91 ± 35	0.2114	11.49 ± 0.56	12.26 ± 2.74	0.3829
t4	1598.21 ± 54	1614.98 ± 41$^{\Delta}$	0.577	12.49 ± 1.55	11.68 ± 1.0$^{\Delta}$	0.3346
t5	1619.74 ± 61	1560.36 ± 40‡	0.1490	12.63 ± 2.15	10.83 ± 1.38‡	0.0732
t6	1607.20 ± 45	1545.13 ± 27$^{\lozenge}$	0.0727	11.79 ± 0.52	12.12 ± 1.08‡	0.5298

$^{\Delta}$… n = 6, ‡… n = 5, $^{\lozenge}$… n = 4

Supplementary Table 4

Supplementary Table 4: Results of the MR relaxometry quality assurance. In the longitudinal study, paravertebral muscle was used as an internal standard for quality assurance of relaxation time estimation. The relaxation times obtained were fairly constant over the entire study period of twelve months. Because muscle was not sufficiently visible in some T1 experiments, data from both sexes were pooled. Unless otherwise stated differently, data of seven animals per group are shown at each time point (data represented as mean +/- standard deviation).

**male**	T2 paravertebral muscle (ms)		**female**	T2 paravertebral muscle (ms)	
time	**control**	**mutant**	time	**control**	**mutant**
t2	20.61 ± 1.46$^{\Diamond}$	19.98 ± 0.87$^{\Diamond}$	t2	19.42 ± 0.89$^{\Diamond}$	19.66 ± 0.46$^{\Diamond}$
t3	20.33 ± 0.41	19.83 ± 0.73	t3	20.22 ± 0.79	19.91 ± 0.72
t4	20.59 ± 1.21	20.07 ± 1.00$^{\Delta}$	t4	19.51 ± 1.17	19.28 ± 1.69$^{\Delta}$
t5	19.75 ± 0.90	19.53 ± 0.91$^{\Delta}$	t5	18.60 ± 0.90	18.60 ± 1.32‡
t6	19.41 ± 0.78	19.36 ± 0.95$^{\Delta}$	t6	19.70 ± 1.33	20.50 ± 0.92‡

$^{\Delta}$… n = 6; ‡… n = 5; $^{\Diamond}$… n = 4

**pooled**	T1 paravertebral muscle (ms)	
time	**control**	**mutant**
t2	1748.67 ± 36	1774.97 ± 33
t3	1793.63 ± 57	1802.81 ± 47
t4	1749.00 ± 65	1771.07 ± 22
t5	1730.75 ± 25	1723.57 ± 46
t6	1755.85 ± 60	1736.18 ± 27

Supplementary Figure 5

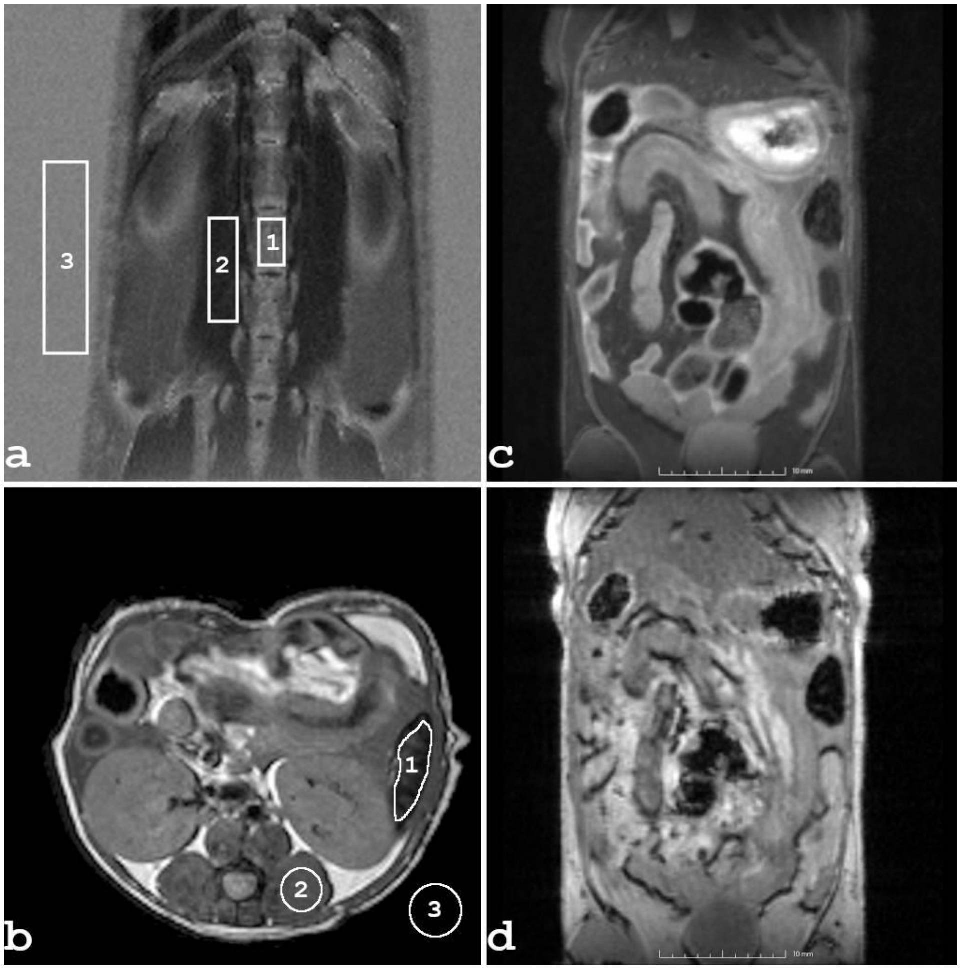

Supplementary Figure 5. Location of the regions of interest (ROIs). **(a)** position of the ROIs for the quantification of the T1 in the vertebral bone marrow (ROI 1), in the paravertebral muscle (ROI 2) and in the background (ROI 3). **(b)** position of the ROIs for the quantification of the T2 in the spleen (ROI 1), in the paravertebral muscle (ROI 2) and in the background (ROI 3). Exemplary images of the applied **(c)** fat saturated 2D-RARE and the **(d)** 3D-FISP protocol acquired from one animal at identical slice position.
